# Cytomegalovirus Infection in a Multiple Sclerosis Patient on Dimethyl Fumarate

**DOI:** 10.1155/crnm/6694528

**Published:** 2025-10-19

**Authors:** Nicolai Larsen

**Affiliations:** Department of Neurology, Naestved Slagelse & Ringsted Hospitals, Region Zealand, Faelledvej 11, Slagelse 4200, Denmark

## Abstract

**Background:**

The association between cytomegalovirus (CMV) infection and multiple sclerosis (MS) remains unclear. CMV infection has been reported in MS patients treated with alemtuzumab, rituximab, and ocrelizumab, but its occurrence in patients receiving dimethyl fumarate (DMF) is less studied.

**Objectives:**

This case report explores the potential relationship between DMF therapy and CMV infection in a relapsing-remitting MS (RRMS) patient, examining whether DMF-induced immune modulation contributed to CMV reactivation or primary infection.

**Results:**

A 29-year-old male with RRMS, on DMF for four years without lymphopenia, developed elevated liver enzymes and splenomegaly. CMV serology showed IgG (60.7 U/L) and IgM (140.0 U/L), with detectable viral DNA (524 IU/mL). Epstein–Barr virus (EBV) IgG was positive, but IgM was negative. Hepatitis A/B, herpes simplex virus (HSV), and varicella tests were negative. DMF was paused for 3 months, and antiviral therapy led to reduced liver enzymes and CMV DNA levels. MS remained stable without new MRI lesions.

**Conclusion:**

DMF therapy may contribute to CMV infection despite the patient's normal lymphocyte counts. It may be beneficial with routinely test with viral panel with patients with progression in MS. Further studies are needed to clarify the risk of CMV infection in DMF-treated MS patients.

## 1. Introduction

The association between cytomegalovirus (CMV) infection and multiple sclerosis (MS) still needs to be studied. Some studies suggest that past CMV and some other herpetiform virus infections have a protective role [1]. In contrast, other data indicate a potential risk of triggering MS [2].

This case presents a patient with relapsing-remitting multiple sclerosis (RRMS) treated with dimethyl fumarate (DMF) in 4 years, who developed CMV infection, as a reactivation or potential primary infection. CMV infection has been reported as a complication of other MS treatments such as alemtuzumab, rituximab, and ocrelizumab, but its association with DMF has been less studied [3–5].

**Figure 1:**
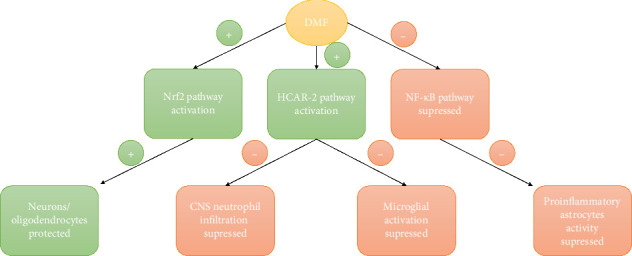
DMF pathways.

DMF has an anti-inflammatory effect through Nrf2-dependent and -independent pathways [6] (Figure 1). Studies suggest that DMF protects neurons and oligodendrocytes, suppresses microglial activation, and mitigates central nervous system (CNS) neutrophil infiltration [6, 7]. These pathways may contribute to explaining DMF's immunosuppressive effects and potential risk of opportunistic infections in MS patients.

## 2. Case Presentation

A 29-year-old male was diagnosed with RRMS after experiencing symptoms onset at the age of 14. He was initially treated with Interferon Beta-1a, switched to Peginterferon Beta-1a, and later transitioned to DMF. Periodically lab control, every 6 months, inclusive routine renal, hepatic, bone marrow, and infection control results were within the normal range (Figure 1). Especially, there were no sign of lymphocytopenia. The magnetic resonance imaging (MRI) showed no new brain or spinal cord lesions. In the neurological examination, the patient presented reduced vision in the upper visual field and increased reflexes in both lower extremities. There were no typical signs of CMV, such as fever, jaundice, or abdominal pain, and the blood sample showed no sign of anemia. A follow-up MRI was performed 3 weeks later and revealed a new lesion in the right corona radiata without diffusion restriction, which prompted a clinical follow-up.

The following month, alanine aminotransferase (ALAT) and lactate dehydrogenase (LDH) progressively worsened (Table 1). ALAT significantly increased, leading to the patient's hospitalization for further evaluation at the hepatological unit. Ultrasound of the abdomen showed splenomegaly and excluded gallbladder stone. Gamma-glutamyl transferase and alkaline phosphatase levels were normal.

Viral serology revealed positive Epstein–Barr virus (EBV) IgG, but not IgM, suggesting a past EBV infection. CMV serology showed IgG at 60.7 IU/mL and IgM at 140.0 IU/mL in serum, confirming an active infection (Table 1). Cerebrospinal fluid (CSF) analysis was negative for CMV and other infection but demonstrated elevated neurofilament light chain and positive oligoclonal bands, consistent with MS activity.

DMF therapy was paused for tree moths. During this period, the patient received antiviral therapy, resulting in reduced liver enzymes and reduction in viral DNA levels. CMV serology showed IgG at 130.0 IU/mL and IgM at 54.7 IU/mL (Table 1). A further follow-up MRI was performed, and no progression of the MS-related lesions was observed. Blood samples were performed weekly, and only mildly elevated liver enzymes were shown. The patient presented no signs of MS progression.

## 3. Discussion

This case highlights the potential risk of interaction between DMF therapy and CMV infection. CMV reactivation is a known complication in patients receiving alemtuzumab, rituximab, or ocrelizumab [3]. However, the association with DMF remains unclear.

Despite DMF's immunomodulatory effect, this patient did not exhibit lymphopenia. Lymphocyte counts remained within normal range during therapy, suggesting the innate immune suppression was likely low to moderate. DMF has an immunomodulatory effect (Figure 1) through Nrf2-dependent and Nrf2-independent pathways [6]. It upregulates anti-inflammatory microglial differentiation and protects neurons [6]. DMF affects the immune system by suppressing CD8+ T-cells and CD4+ T-cells and increasing regulatory T-cells (Tregs) [6, 8]. This may indirectly affect the patient's humoral response and ability to control latent viral infections such as CMV.

However, CMV can activate Nrf2, which DMF also targets [9]. This could contribute to virus reactivation under DMF therapy. In this case, the patient showed CMV IgG, IgM, and CMV DNA, indicating either reactivation or primary infection.

DMF therapy was paused for 3 months during antiviral treatment. In this period, the patient's liver enzymes were downregulated and the CMV DNA levels were also reduced indicating effective viral control. The temporary pause of DMF may have contributed to an improved immune system response, aiding the CMV management. There was no clinical sign of MS progression during the treatment break, which highlights the need to balance the infection.

Progressively elevated liver enzymes were the primary sign of the infection. CMV usually affects the liver, leading to mononucleosis-like symptoms such as fever, abdominal pain, jaundice, spleen, and hepatomegaly [10]. Although the exact association between DMF therapy and CMV infection is unclear, the CNS remained unaffected by CMV, as the CSF analysis was negative for viral infection despite a new white-matter lesion was described on brain MRI.

## 4. Conclusion

In this case, an association between DMF, immunosuppression, and viral infection was shown. It may be beneficial to routinely test viruses with a panel, particularly in patients presenting with unexplained symptoms and progression in MS. Larger studies are needed to study the risk of CMV infection in patients treated with DMF.

## Figures and Tables

**Table 1:** Clinical data presentation.

Parameter	Initial values	CMV infection	Post-treatment
Neurological findings	Reduced visual upper field. Increased reflexes in both lower extremities.	Visual field deficient, increased reflexes in both under extremities.	Stable, no new CNS symptoms.
MRI findings	No new lesions.	New lesion in right corona radiata (no diffusion restriction).	No change.
Liver function tests	Normal ALAT, ASAT, LDH.	ALAT↑ (510 U/L), LDH↑ (290 U/L), ASAT↑ (180 U/L).	Gradual normalizing.
Leukocytes	Normal	Normal	Normal
Lymphocytes	Normal	Normal	Normal
C-reactive protein	Normal	Normal	Normal
Spleen size	Unknown.	Splenomegaly (15.7 cm).	Unknown.
CMV IgG/IgM	Unknown.	IgG (60.7 U/L), IgM (140.0 U/L).	IgG (130.0 U/L), IgM (54.7 U/L).
CMV DNA/RNA load	Unknown.	524 (IU/mL).	85 (IU/mL).
CFS virus	Unknown.	No infection detected.	No change.
CSF oligoclonal bands	Positive.	Positive.	Stable.
Neurofilament light levels	Unknown.	Elevated (171 ng/L).	Unknown.

## Data Availability

Data used to support the findings of this study are available on request due to privacy/ethical restrictions.
